# Myeloid-Derived Suppressor Cells as Key Players and Promising Therapy Targets in Prostate Cancer

**DOI:** 10.3389/fonc.2022.862416

**Published:** 2022-07-04

**Authors:** Izabela Siemińska, Jarek Baran

**Affiliations:** ^1^ Department of Clinical Immunology, Jagiellonian University Medical College, Cracow, Poland; ^2^ University Centre of Veterinary Medicine, Jagiellonian University - University of Agriculture, Cracow, Poland

**Keywords:** prostate cancer, myeloid-derived suppressor cells, immunosuppression, immunotherapy, anti-tumor immune response

## Abstract

Prostate cancer (PC) is the second most often diagnosed malignancy in men and one of the major causes of cancer death worldwide. Despite genetic predispositions, environmental factors, including a high-fat diet, obesity, a sedentary lifestyle, infections of the prostate, and exposure to chemicals or ionizing radiation, play a crucial role in PC development. Moreover, due to a lack of, or insufficient T-cell infiltration and its immunosuppressive microenvironment, PC is frequently classified as a “cold” tumor. This is related to the absence of tumor-associated antigens, the lack of T-cell activation and their homing into the tumor bed, and the presence of immunological cells with regulatory functions, including myeloid-derived suppressor cells (MDSCs), regulatory T cells (Treg), and tumor-associated macrophages (TAMs). All of them, by a variety of means, hamper anti-tumor immune response in the tumor microenvironment (TME), stimulating tumor growth and the formation of metastases. Therefore, they emerge as potential anti-cancer therapy targets. This article is focused on the function and role of MDSCs in the initiation and progression of PC. Clinical trials directly targeting this cell population or affecting its biological functions, thus limiting its pro-tumorigenic activity, are also presented.

## Prostate Cancer—Epidemiology

Prostate cancer (PC) is the most common, after lung cancer, malignancy in men—in 2020, more than 1.4 million new cases of PC were diagnosed worldwide (1, 2). Advanced age, race, and ethnicities such as African descent and family history are well-established risk factors of PC (3–6). Additionally, a higher incidence of PC has been associated with a diet rich in saturated animal fat and red meat, low intake of fruits/vegetables, obesity, hyperglycemia, lack of physical activity, prostate inflammation, as well as exposure to chemicals or ionizing radiation (6–8). The most common genetic predispositions for PC development are related to aberrations of the *PTEN* tumor suppressor gene. Inactivation of *PTEN* by deletion or mutations is identified in ∼20% of primary PC and as many as 50% of advanced castration-resistant tumors (9). The role of the immune system and prostatitis in PC development was also confirmed, indicating that inflammatory mediators may promote prostatic carcinogenesis *via* inhibition of apoptosis, promotion of cell proliferation, and even loss of the tumor suppressor genes (10). Importantly, not only the local, prostate inflammation, but also systemic reaction associated with chronic inflammatory diseases, including asthma and allergies, are associated with the higher risk of PC (11).

Most of the patients develop a low-risk neoplasm (12); however, approximately 15% of men with localized PC present with high-risk tumors, which will progress, metastasize, and finally result in death (13). In men with advanced metastatic prostate cancer (mPC), hormonal–androgen deprivation therapy is a method of choice with a good response rate. In some patients, however, the mPC will evolve into metastatic castration-resistant prostate cancer (mCRPC) (14). While a radical prostatectomy may be beneficial for patients with high-risk PC (15), only multimodal treatment, including surgery, radiation, and systemic therapy, gives the best chance for a long-term progression-free outcome (13). Nowadays, immunotherapy options, including anti-PC vaccines, e.g., Sipuleucel-T (Provenge), and the use of immune checkpoint inhibitors (anti-CTLA-4 and anti-PD-1/PD-L1 monoclonal antibodies or antagonists) further improve the effectiveness of the PC treatment (16).

PC is often considered a “cold” tumor, meaning that due to the reduced or complete lack of T-cell infiltration, e.g., because of the missing tumor-associated antigens, lack of T-cell activation and their homing into the tumor bed, and local immunosuppression, it does not trigger a strong immune response. This term emphasizes the role of the immune system in PC progression (16, 17). Studies indicate that regulatory T cells (Tregs) and other cell populations, namely, myeloid-derived suppressor cells (MDSCs; attracted to TME by low-grade chronic inflammatory signals) and tumor-associated macrophages (TAMs) (17), are mainly responsible for the immunosuppression observed in PC (18). Among them, MDSCs emerge as potential therapeutic targets (19).

## Myeloid-Derived Suppressor Cells—Their Origin and Activity

The term “myeloid-derived suppressor cells” has been used in the literature since 2007; however, the history of these cells dates back to the early 20th century, when it was shown that cancer is often accompanied by extra-medullary hematopoiesis (EMH) and neutrophilia (20, 21). These immature leukocytes were further characterized by their suppressive activity and called myeloid suppressor cells (MSC) (22). This term was further changed to MDSCs (22), and although current, the progress in resolution techniques, including a high-dimensional single-cell analysis, has raised concerns regarding the development and activation state of MDSCs (23); it is still accepted that MDSCs represent a heterogeneous population of immature myeloid cells, promptly expanding during pathological conditions, including infection, inflammation, and cancer (24). With respect to their origin, MDSCs have been divided into two main subsets—monocytic (Mo-MDSCs) and granulocytic or polymorphonuclear (PMN-MDSCs). Recently, a third population of the so-called early-stage MDSCs (e-MDSCs) was also described (25). In cancer, the accumulation of MDCSs is inseparably related to the production of pro-inflammatory mediators by the tumor microenvironment (TME), which activate and drive their suppressive activity (26). The immunosuppressive mechanisms developed by MDSCs are diverse and may include arginase-1 (ARG1) and inducible nitric oxide synthase (iNOS) activity; secretion of TGFβ, IL-10, and cyclooxygenase-2 (COX-2); and depletion of tryptophan by indoleamine 2,3-dioxygenase (IDO) (27). Although the immunosuppressive nature and the induction of antigen-specific T-cell tolerance is common for all the MDSCs subsets (28), they differ in the mechanism of action. In this context, Mo-MDSCs suppress T-cell response in both an antigen-specific and an unspecific manner, utilizing the mechanisms associated with iNOS activity and production of nitric oxide (NO) (29, 30). In contrast, PMN-MDSCs suppress immune response primarily in an antigen-specific manner, using the STAT3-mediated mechanisms of NADPH-oxidase and ARG1 activities (31). PMN-MDSCs store ARG1 in the granules and release it to the extracellular milieu, leading to the local depletion of L-arginine, affecting T-cell functionality. Both MDSCs subsets release ROS, which are essential for their immunosuppressive activity, and for retaining their undifferentiated status. Numerous studies confirmed the interplay between chronic inflammatory factors and expansion of MDSCs (24, 32). The transcription factor STAT3 plays a central role in the generation and functioning of MDSCs (33–35). Various cytokines, including IL-6, IL-1β, IL-10, GM-CSF, and VEGF, secreted mainly in the TME by tumor cells (26), are involved in the activation of pSTAT3. Conversely, chronic inflammation is associated with the initiation and progression of the tumor (10). In this context, chemokines and their receptors, e.g., CCL2/CCL12-CCR2, CXCL5/2/1-CXCR2, CCL3/4/5-CCR5, CCL15-CCR1, and CXCL8-CXCR1/2, are relevant for a rapid progression of PC and the recruitment of MDSCs (36, 37). PC patients were shown to have higher MDSCs infiltration than those with a benign prostate hyperplasia (38). Therefore, the role of inflammation in the development and expansion of MDSCs, and hence in PC progression, is unquestionable.

## Expansion of MDSCs in PC

Studies with the use of *PTEN* KO murine PC model documented that lack of this gene was associated with upregulated inflammatory response (enhanced production of CSF-1 and IL-1β), and an extensive MDSCs tumor infiltration (39). Another mechanism involved in the recruitment of MDSCs in PC could be linked to the Hippo–YAP signaling. This pathway, relevant for the regulation of cell proliferation and apoptosis, is often deregulated in human solid tumors and associated with enhanced cancer cell proliferation (40). In PC, the hyperactivated Hippo–YAP signaling causes the upregulation of CXCL5 in cancer cells, which promotes the MDSCs recruitment *via* the CXCL5–CXCR2 axis (41, 42). The recruitment of MDSCs to the tumor mass may also benefit from the tumor-related hypoxia. This is supported by the observation that the hypoxia-targeted therapy may lead to a long-lasting decrease in the accumulation of MDSCs in the tumor (43). A significant role in the recruitment of MDSCs to PC has also been assigned to chromodomain helicase DNA-binding protein 1 (CHD1), an essential tumor suppressor (44). Its depletion was found in 29.7% of cases in African Americans, and 11.0% of European PC patients (45). It has been shown that CHD1 deficiency may recruit MDSCs *via* an IL-6-dependent mechanism (46). Interestingly, a positive correlation between CHD1 and CD15 expression (a surface marker of PMN-MDSCs) in PC was also documented (46).

A growing list of evidence suggests that miRNA carried by tumor-derived extracellular vesicles (TEVs) may also play a role in the generation of MDSCs in many types of cancer (47–49). Although there are no data confirming such a role of EV miRNA in PC, some miRNAs already shown as relevant in the induction of MDSCs in other cancers have also been considered for PC (50).

**Figure 1:**
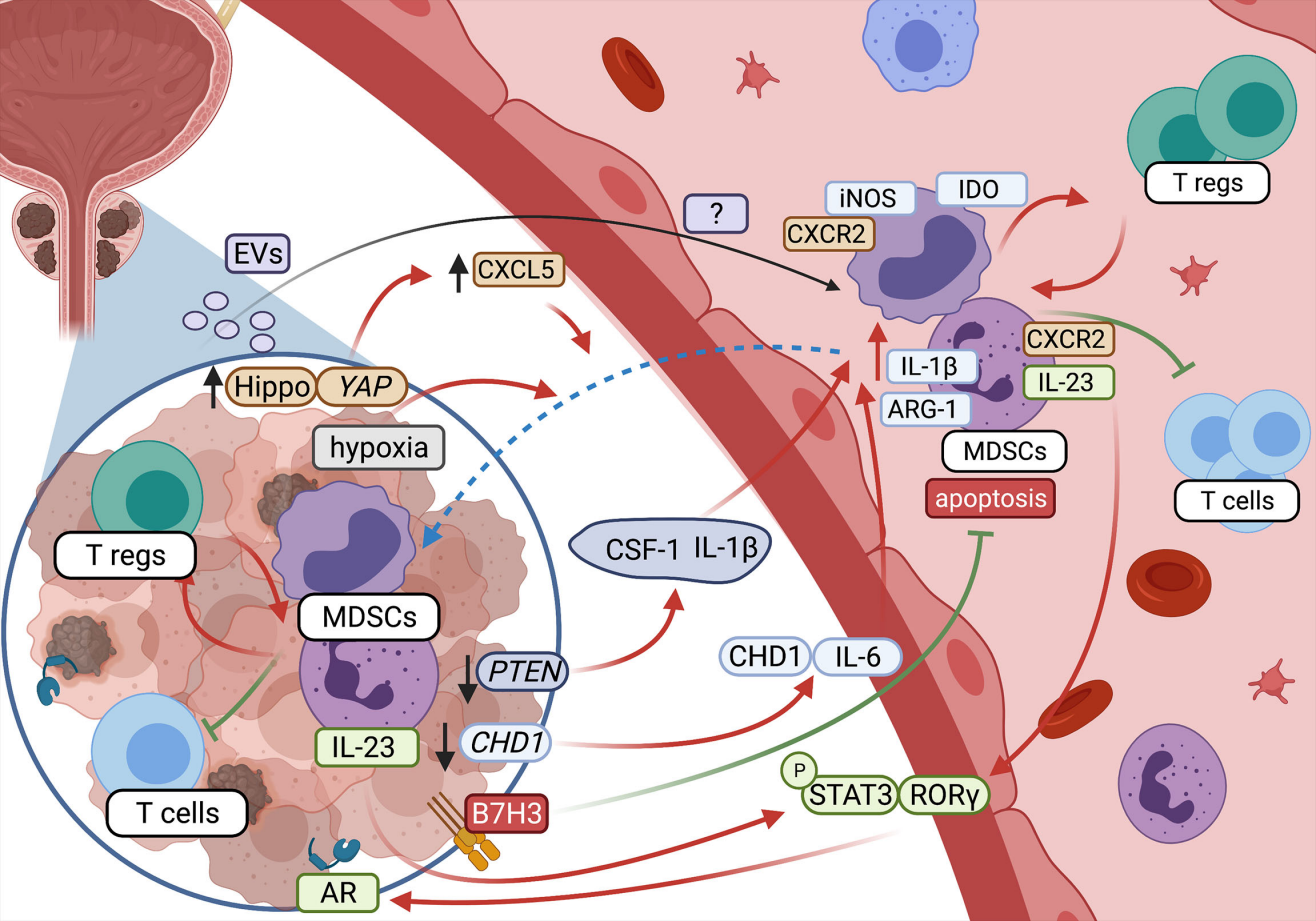
Crosstalk between MDSCs and tumor microenvironment in prostate cancer (created with BioRender.com).

The crosstalk between MDSCs and the TME in PC is schematically presented in **Figure 1**.

## Role of MDSCs in PC Development and Progression

In various cancers, the level of tumor-infiltrating MDSCs has been proposed as a prognostic marker (51, 52). In PC, however, such data are scarce and refer mainly to the *PTEN* mouse model (39). In contrast, there are observations that the MDSCs’ blood level could be a useful parameter for monitoring the disease burden in PC, allowing researchers to distinguish between metastatic cancer, localized PC, and cancer-free men (53). Additionally, circulating MDSCs correlate well with PSA level and metastasis (33, 54). The pivotal role of MDSCs in the development and progression of PC was further confirmed in randomized clinical studies showing that the increased level of MDSCs after the treatment is associated with the overall worse patients’ survival (55, 56). Moreover, in a mouse model of PC, the lung infiltration by MDSCs was associated with the formation of lung metastases (57). However, what type of MDSCs subpopulation is pivotal and prevalent in PC remains controversial, mainly due to the lack of reproducibility and standardization of such research. The work showing MDSCs as a negative prognostic marker in mCRPC indicates only blood Mo-MDSCs as relevant (58). Furthermore, in patients with mCRPC, a positive correlation between Mo-MDSCs and Treg cells has been described (58), suggesting a mutual positive feedback loop (59). Generally, most of the studies in PC have focused on Mo-MDSCs rather than on PMN-MDSCs (55, 58, 60). Even early reports on circulating immunosuppressive cells in patients with PC were concentrated on CD14+HLA-DR^low/-^ monocytes (54). This may result from the fact that Mo-MDSCs are more frequent in peripheral blood than PMN-MDSCs (61, 62). Another reason could be the fact that, in many studies, a cryopreserved material was used (63), affecting the recovery of PMN-MDSCs (64). Recently, Wen et al. documented infiltration of the primary prostate tumor by cells referred to as PMN-MDSCs (65); however, the markers used for their identification did not allow researchers to distinguish them from the population of tumor-associated neutrophils (TANs) (25). In this context, the phenotype definition of circulating blood PMN-MDSCs seems to be more reliable, but still, this should be further confirmed by functional tests that document the immunosuppressive nature of these cells (25).

Studies in PC showed that Mo-MDSCs and PMN-MDSCs are transcriptomically different (61), pointing out the ARG1 as typical for PMN-MDSCs (66) and iNOS or IDO for Mo-MDSCs (58, 60). Moreover, PMN-MDSCs can exert their immunosuppressive action also by the release of neutrophil elastase (NE), which was shown to stimulate the proliferation, migration, and invasion of cancer cells both *in vitro* and *in vivo* in a mouse model of PC (67, 68).

It is proposed that, in PC, the tumor-infiltrating PMN-MDSCs express upregulated IL-1β and IL-23a (66). Although the IL-1β-restrained antitumor immunity was described before for other tumors (69), the secretion of IL-23 by PMN-MDSCs so far has been documented only for PC. In this context, it was shown that IL-23 preserves the androgen receptor’s (AR) functionality, enabling survival and proliferation of PC in the androgen-deprived environment. The same mechanism is postulated as a driving force in the development of castration resistance (40). However, castration resistance may also be related to the secretion of IL-8 and subsequent tumor infiltration by PMN-MDSCs (66).

## Targeting MDSCs in PC

Due to a lack of, or insufficient T-cell infiltration and immunosuppressive microenvironment in PC, there is a need to design new therapies that could “turn up the heat on the cold immune microenvironment” (17), to enhance the local anti-tumor immune response (16). Radiation *per se* has been found to activate the immune response (70); however, studies using the animal models of PC revealed that radiotherapy induces a rapid increase in the tumor-infiltrating MDSCs (71). Our previous studies showed that surgery or hormonal therapy alone did not reduce the level of circulating Mo-MDSCs in PC patients (62). In this context, in addition to the standard treatment, immunotherapy (72) or dietary strategies (73) are implemented, targeting cells with immunosuppressive potential, including MDSCs. One of the major challenges in targeting human MDSCs is their heterogeneous nature, e.g., differences in phenotype and mechanisms of suppression. A type of “universal” approach, covering the above aspects, may be the use of gemtuzumab ozogamicin, a calicheamicin-conjugated anti-CD33 humanized monoclonal antibody, already approved to treat a subset of patients with acute myeloid leukemia, which has also been highly effective against MDSCs in many solid tumors, including PC *in vitro* (61).

Clinically, MDSCs may be targeted by different approaches, including, e.g., inhibition of MDSCs expansion, MDSCs depletion, induction of their differentiation, functional inhibition, or multifactorial treatment. The clinical trials concerning all these potentially therapeutic strategies in PC have been described below **Table 1**.

**Table 1:** Clinical trials targeting MDSCs in PC patients.


No.	Title	Condition or disease	Interventions	Mechanism of action	Trial number	Status
	**Inhibition of MDSC expansion**
1	Combination Study of AZD5069 and Enzalutamide. (ACE)	Metastatic Castration-Resistant Prostate Cancer	CXCR2 antagonist + enzalutamide	CXCR2 antagonist may block recruitment of MDSCs to the tumor (41)	NCT03177187	Recruiting
2	Immediate Prostatectomy vs. Cabozantinib Followed by Prostatectomy in Men with High-Risk Prostate Cancer (SPARC)	Prostate Cancer Prostate Cancer Adenocarcinoma Non-Metastatic	Cabozantinib (small molecule inhibitor of tyrosine kinase receptor) + Radical prostatectomy	Cabozantinib may reduce the tumor infiltration by MDSCs (76)	NCT03964337	Recruiting
3	Soy Bread Diet in Improving Immune Function in Participants With Prostate Cancer	Prostate Adenocarcinoma	Dietary intervention	Soy bread isoflavones may reduce pro-inflammatory cytokines and MDSCs level (77)	NCT03654638	Recruiting
**MDSC depletion**						
4	Enzalutamide and Decitabine in Treating Patients with Metastatic Castration Resistant Prostate Cancer	Metastatic Castration-Resistant Prostate Cancer	Decitabine (nucleic acid synthesis inhibitor)	Decitabine (5-aza-2′-deoxycytidine), a hypomethylating agent with the ability to selectively deplete Mo-MDSCs (80)	NCT03709550	Withdrawn
**Stimulation of MDSC differentiation**						
5	Trial of Curcumin to Prevent Progression of Low-risk Prostate Cancer Under Active Surveillance	Prostate Cancer	Curcumin	Curcumin may promote the differentiation of MDSCs (81)	NCT03769766	Recruiting
6	White Button Mushroom Soup for the Reduction of PSA in Patients with Biochemically Rec or Therapy Naive Fav Risk Prostate CA	Prostate Adenocarcinoma PSA Failure PSA Progression Recurrent Prostate Carcinoma Stage I Prostate Cancer stage IIA–C, III A, C	White Button Mushroom (WBM) Extract	WBM powder as a source of β-glucan may induce MDSC differentiation to antigen-presenting cells (83) and reduce the number of circulating MDSCs (84)	NCT04519879	Recruiting
**Inhibition of MDSCs induced suppressive mechanisms**						
7	Abiraterone Acetate in Combination with Tildrakizumab (ACTIon)	Metastatic Castration-Resistant Prostate Cancer	Abiraterone Acetate (selective inhibitor of CYP17) + Tildrakizumab (anti-IL-23)	Tildrakizumab (anti-IL-23 mAb), alters the production of IL-23 in PC, has the potential to affect castration resistance caused by MDSCs (41)	NCT04458311/2019-003485-40	Recruiting
8	A Trial of Ipatasertib in Combination with Atezolizumab (IceCAP)	Solid Tumor Glioblastoma Multiforme Prostate Cancer Metastatic	Ipatasertib (inhibitor of all three isoforms of protein kinase AKT) + Atezolizumab (anti-PD-L1)	Atezolizumab (anti-PD-L1 monoclonal antibodies)—a checkpoint inhibitor on MDSCs (86)	NCT03673787	Recruiting
9	A phase I/II basket trial evaluating a combination of Metronomic Oral Vinorelbine plus anti-PD-L1/anti-CTLA4 Immunotherapy in patients with advanced solid tumors.	Patients with locally advanced or metastatic solid tumors	Vinorelbine (cytostatic) Durvalumab (anti-PD-L1) + Tremelimumab (anti-CTLA-4)	Anti-PD-L1 and anti-CTLA-4 checkpoint inhibitors on MDSCs (86)	2017-001857-14	Ongoing
**Multifactorial action: inhibition of MDSC expansion and blocking their suppressive activity**						
10	A Phase 1b/2, Open-Label, Multicenter Study Assessing the Safety, Tolerability, Pharmacokinetics, and Preliminary Anti-tumor Activity of MEDI4736 in Combination with AZD9150 or AZD5069 in Patients with Advanced Solid Malignancies and Subsequently Comparing AZD9150 and AZD5069 Both as Monotherapy and in Combination with MEDI4736 as Second-Line Treatment in Patients with Recurrent and/or Metastatic Squamous Cell Carcinoma of the Head and Neck.	Part A: Advanced solid tumor Part B: Recurrent and/or spreading tumor of Head and Neck	MEDI4736 (Durvalumab- anti-PD-L1) + AZD9150 (Danvatirsen-STAT3 inhibitor) + AZD5069 (CXCR2 antagonist)	Combination of STAT3 inhibitor, selective CXCR2 antagonist, and PD-L1 inhibitor, where each of them has the potential to inhibit MDSCs activity (41, 60, 86)	2015-002525-19	Restarted or completed depending on the country
11	Phase II Trial of EP4 Receptor Antagonist in Advanced Solid Tumors	Prostate Cancer Non-Small Cell Lung Cancer Breast Cancer	Grapiprant + Gemcitabine	Gemcitabine may reduce the level of MDSCs (87), whereas grapiprant, a PGE2-receptor inhibitor, may block induction of MDSCs (88) and their suppressive effect (26)	NCT02538432	Withdrawn

### Inhibition of MDSCs Expansion

Currently, there are three registered clinical trials, aiming at the inhibition of MDSCs expansion in PC. As mentioned, chemokines and their receptors are pivotal for the recruitment of MDSCs and the rapid progression of PC (36, 37); therefore, targeting the chemokine receptors or the use of chemokine inhibitors seems to be a promising form of immunotherapy in PC (74). One of the ongoing clinical trials (NCT03177187) seems to verify this hypothesis by using the CXCR2 antagonist AZD5069 in combination with enzalutamide—the androgen receptor’s antagonist in patients with mCRPC (75). An important additional factor associated with MDSCs expansion is VEGF (26); thus, administration of cabozantinib (a small-molecule inhibitor of tyrosine kinase receptor, including the VEFG pathway) followed by radical prostatectomy vs. prostatectomy alone (NCT03964337) is being tested in men with high-risk PC. Moreover, cabozantinib has already shown inhibitory effects on MDSCs (76). Another trial concerning dietary intervention, NCT03654638, is focused on soy bread, containing isoflavones, which were shown to reduce the level of pro-inflammatory cytokines and MDSCs (77).

### MDSCs Depletion

MDSCs isolated from both mice and humans display elevated levels of STAT3, while inhibition of its pathway resulted in enhanced antitumor activity (28, 78). Circulating Mo-MDSCs maintain high levels of STAT3 until they reach the tumor, where hypoxia induces its rapid downregulation, causing differentiation of MDSCs to TAMs (79). STAT3 regulates the expression of the main factors of MDSCs activity, e.g., IDO, ARG1, IL-6, IL-10, IL-1β, and VEGF, among others, suggesting this pathway as an attractive therapeutic option (26). In this context, a fungal-derived pSTAT3 inhibitor, galiellalactone, was recently assessed for its ability to prevent PC-induced generation of MDSCs *in vitro* (53). In keeping with this, the clinical trial NCT03709550, aiming at testing decitabine (5-aza-2′-deoxycytidine), a hypomethylating agent with the ability to selectively deplete Mo-MDSCs, in mCRPC patients was implemented (80).

### Inhibition of MDSCs Differentiation

Another therapeutic option involves a controlled differentiation of MDSCs towards the M1 anti-tumor macrophages with the use of curcumin (81). This approach will be considered in the recruiting clinical trial (NCT03769766). A similar approach will be used in the phase I clinical study in patients with biochemically recurrent PC, testing the effectiveness of the white button mushroom (WBM) extract containing β-glucan (NCT04519879). β-glucans, the most abundant carbohydrates found in yeast and mushrooms (82), may induce MDSCs differentiation to antigen-presenting cells, eliminating their suppressive abilities (83). The rationale for this concept was additionally grounded on the preclinical data showing that dietary WBM powder reduced not only the frequency of circulating MDSCs but also the level of prostate-specific antigen (PSA) (84).

### Inhibition of MDSCs Induced Suppressive Circuits

There is also a possibility to inhibit some of the MDSCs-induced suppressive mechanisms operating in PC. One of such approaches is represented by a combination of abiraterone, a novel hormone therapy available for CRPC (85), and tildrakizumab (anti-IL-23 mAb) (NCT04458311), altering the production of IL-23 and therefore having a potential to target the MDSCs function specific for PC (41). In another clinical trial, a combination of ipatasertib (inhibitor of all three isoforms of protein kinase AKT, which blocks the PI3K/AKT signaling pathway—a key driver of cancer cell growth and proliferation in PC), atezolizumab (anti-PD-L1 monoclonal antibodies)—a checkpoint inhibitor on MDSCs (86), and docetaxel (NCT03673787) will be tested in patients with mCRPC. Currently, in Europe, there is one registered clinical trial focused on blocking the MDSCs function in PC patients (no. 2017-001857-14). It tests the combination of vinorelbine, a cytostatic drug, and two checkpoint inhibitors, durvalumab and tremelimumab, which are anti-PD-L1 and anti-CTLA-4 mAb, respectively.

### Multifactorial Intervention: Inhibition of MDSCs Expansion and Blocking Their Suppressive Activity

Combinations of both the inhibition of MDSCs expansion and blocking their suppressive activity provide the opportunity for multifactorial interventions with potential better therapeutic effectiveness. One of such trials tests the combination of STAT3 inhibitor (AZD9150), a selective CXCR2 antagonist (AZD5069), and the PD-L1 inhibitor (MEDI4736) (no. 2015-002525-19), where each can inhibit either MDSCs expansion or function. Another drug combination that is being tested is gemcitabine and RQ-00000007 (grapiprant), where gemcitabine inhibits MDSCs expansion (87), while grapiprant—an inhibitor of PGE2-receptor—reduces the differentiation, expansion, and suppressive activities of Mo-MDSCs (88), confirming its role in MDSCs functioning (26).

### Potential New Targets

Despite a wide scope of the ongoing clinical research, there are other available potential therapeutic options targeting MDSCs in PC. One, yet unexplored route, concerns the angiotensin-converting enzyme (ACE)–angiotensin pathway, where the overexpression of ACE in monocytic cells was shown to reduce the generation of MDSCs (89), while angiotensin was able to reduce the tumor malignancy in PC (90). Nowadays, during the SARS-CoV-2 pandemic, this pathway, however, takes on a quite different significance. However, other forms of angiotensin may impact the biological properties of PC cells by modulating inflammatory reaction, or even genes, including downregulation of HIF1a and upregulation of CDH-1 (91) expression, both associated with MDSCs recruitment. Another potential approach involves estrogen, used previously in PC therapy (92). The combined therapy, linking activation of estrogen receptor β (ERβ) and the checkpoint inhibitor anti-PD-1 mAb, diminishes MDSCs infiltration in mouse models of colorectal and breast cancer (93). Interestingly, apoptosis and/or differentiation of PC cells may be promoted during the ERβ activation (94). Additionally, studies confirmed the benefits of ERβ activation in androgen-dependent CRPC, decreasing the viability of the tumor cells (95). Also, ARG1 is a potential therapeutic target in PC, and its inactivation through STAT3 inhibition was already confirmed (34). The ongoing clinical trials aiming at targeting MDSCs may be a trigger for more frequent use of immunotherapy in combination with other forms of PC treatment.

## Conclusion

Although the first observations reporting the negative role of MDSCs in antitumor responses in PC date back from the beginning of the 21st century, the last decade saw an upsurge of studies indicating their mechanisms of action and clinical relevance (96). Although several questions remain unanswered, the role of MDSCs in the development and progression of PC seems unquestionable, suggesting their potential as a therapeutic target. Hence, the implementation of the combination therapy, e.g., radiotherapy and immunotherapy, targeting both the tumor and MDSCs in PC seems crucial. Such therapy may increase the frequency of the abscopal response, which is a phenomenon associated with tumor shrinkage, occurring not only locally at the site of the treatment but also in other locations, where the tumor has already spread (97).

## Author Contributions

IS wrote the draft version of the manuscript. JB revised and edited the final version of the manuscript. All authors contributed to the article and approved the submitted version.

## Funding

The authors declare and acknowledge financial support from EU H2020-MSCA-RISE-2017 program - grant “CANCER” (GA 777682).

## Conflict of Interest

The authors declare that the research was conducted in the absence of any commercial or financial relationships that could be construed as a potential conflict of interest.

## Publisher’s Note

All claims expressed in this article are solely those of the authors and do not necessarily represent those of their affiliated organizations, or those of the publisher, the editors and the reviewers. Any product that may be evaluated in this article, or claim that may be made by its manufacturer, is not guaranteed or endorsed by the publisher.

## References

[1] Sung H Ferlay J Siegel RL Laversanne M Soerjomataram I Jemal A. Global Cancer Statistics 2020: GLOBOCAN Estimates of Incidence and Mortality Worldwide for 36 Cancers in 185 Countries. CA: A Cancer J Clin (2021) 71:209–49. doi: 10.3322/caac.21660 33538338

[2] Rawla P. Epidemiology of Prostate Cancer. World J Oncol (2019) 10:63. doi: 10.14740/WJON1191 31068988 PMC6497009

[3] Rebbeck TR. Prostate Cancer Genetics: Variation by Race, Ethnicity, and Geography. Semin Radiat Oncol (2017) 27:3. doi: 10.1016/J.SEMRADONC.2016.08.002 27986209 PMC5175208

[4] Pienta KJ Esper PS. Risk Factors for Prostate Cancer. Ann Internal Med (1993) 118:793–803. doi: 10.7326/0003-4819-118-10-199305150-00007 8470854

[5] Bostwick DG Burke HB Djakiew D Euling S Ho SM Landolph J. Human Prostate Cancer Risk Factors. Cancer (2004) 101:2371–490. doi: 10.1002/CNCR.20408 15495199

[6] Dagnelie PC Schuurman AG Goldbohm RA van den Brandt PA. Diet, Anthropometric Measures and Prostate Cancer Risk: A Review of Prospective Cohort and Intervention Studies. BJU Int (2004) 93:1139–50. doi: 10.1111/J.1464-410X.2004.04795.X 15142129

[7] Kolonel LN. Fat, Meat, and Prostate Cancer. Epidemiol Rev (2001) 23:72–81. doi: 10.1093/OXFORDJOURNALS.EPIREV.A000798 11588857

[8] Wolk A. Diet, Lifestyle and Risk of Prostate Cancer. Acta Oncol (Stockholm Sweden) (2005) 44:277–81. doi: 10.1080/02841860510029572 16076700

[9] Jamaspishvili T Berman DM Ross AE Scher HI de Marzo AM Squire JA. Clinical Implications of PTEN Loss in Prostate Cancer. Nat Rev Urol (2018) 15:222–34. doi: 10.1038/NRUROL.2018.9 PMC7472658 29460925

[10] Jiang J Li J Zhang Y Zhu H Liu J Pumill C. The Role of Prostatitis in Prostate Cancer: Meta-Analysis. PLos One (2013) 8:85179. doi: 10.1371/JOURNAL.PONE.0085179 PMC3877315 24391995

[11] Beckmann K Russell B Josephs D Garmo H Haggstrom C Holmberg L. Chronic Inflammatory Diseases, Anti-Inflammatory Medications and Risk of Prostate Cancer: A Population-Based Case-Control Study. BMC Cancer (2019) 19:1–9. doi: 10.1186/S12885-019-5846-3/TABLES/5 31226970 PMC6588859

[12] Punnen S Cooperberg MR. The Epidemiology of High-Risk Prostate Cancer. Curr Opin Urol (2013) 23:331–6. doi: 10.1097/MOU.0B013E328361D48 23619582

[13] McKay RR Feng FY Wang AY Wallis CJD Moses KA. Recent Advances in the Management of High-Risk Localized Prostate Cancer: Local Therapy, Systemic Therapy, and Biomarkers to Guide Treatment Decisions. ASCO Educ Book (2020) 40:241–52. doi: 10.1200/EDBK_279459 PMC10182417 32412803

[14] Shelley M Harrison C Coles B Stafforth J Wilt T Mason M. Chemotherapy for Hormone-Refractory Prostate Cancer. Cochrane Database Syst Rev (2006) 4. doi: 10.1002/14651858.CD005247.PUB2 PMC12399875 17054249

[15] Ghodoussipour S Cacciamani GE Luis A Abreu C. Radical Prostatectomy for High-Risk Prostate Cancer | Opinion: No. Int Braz J Urol (2019) 45(3):428–34. doi: 10.1590/S1677-5538.IBJU.2019.03.03 PMC6786100 31149790

[16] Fay EK Graff JN. Immunotherapy in Prostate Cancer. Cancers (2020) 12:1–17. doi: 10.3390/CANCERS12071752 PMC7409298 32630247

[17] Stultz J Fong L. How to Turn Up the Heat on the Cold Immune Microenvironment of Metastatic Prostate Cancer. Prostate Cancer Prostatic Dis (2021) 24:697–717. doi: 10.1038/s41391-021-00340-5 33820953 PMC8384622

[18] Sfanos KS Bruno TC Maris CH Xu L Thoburn CJ Demarzo AM. Phenotypic Analysis of Prostate-Infiltrating Lymphocytes Reveals TH17 and Treg Skewing. Clin Cancer Res (2008) 14:3254–61. doi: 10.1158/1078-0432.CCR-07-5164 PMC3082357 18519750

[19] Li X Zhong J Deng X Guo X Lu Y Lin J. Targeting Myeloid-Derived Suppressor Cells to Enhance the Antitumor Efficacy of Immune Checkpoint Blockade Therapy. Front Immunol (2021) 0:754196. doi: 10.3389/FIMMU.2021.754196 PMC8727744 35003065

[20] Sonnenfeld A. Leukamische Reaktiones Bei Carcinoma. Z f Klin Med 1929) 111:108.

[21] Talmadge JE Gabrilovich DI. History of Myeloid Derived Suppressor Cells (MDSCs) in the Macro-and Micro-Environment of Tumour-Bearing Hosts. Nat Rev Cancer (2013) 13:739–52. doi: 10.1038/nrc3581 PMC4358792 24060865

[22] Gabrilovich DI Bronte V Chen S-H Colombo MP Ochoa A Ostrand-Rosenberg S. The Terminology Issue for Myeloid-Derived Suppressor Cells. Cancer Res (2007) 67:425–5. doi: 10.1158/0008-5472.CAN-06-3037 PMC1941787 17210725

[23] Hegde S Leader AM Merad M. MDSCs: Markers, Development, States, and Unaddressed Complexity. Immunity (2021) 54:875–84. doi: 10.1016/J.IMMUNI.2021.04.004 PMC8709560 33979585

[24] Sinha P Chornoguz O Clements VK Artemenko KA Zubarev RA Ostrand-Rosenberg S. Myeloid-Derived Suppressor Cells Express the Death Receptor Fas and Apoptose in Response to T Cell-Expressed FasL. Blood (2011) 117:5381–90. doi: 10.1182/blood-2010-11-321752 PMC3109712 21450901

[25] Bronte V Brandau S Chen S Colombo MP Frey AB Greten TF. Recommendations for Myeloid-Derived Suppressor Cell Nomenclature and Characterization Standards. Nat Commun (2016) 7:1–10. doi: 10.1038/ncomms12150 PMC4935811 27381735

[26] Parker KH Beury DW Ostrand-Rosenberg S. Myeloid-Derived Suppressor Cells: Critical Cells Driving Immune Suppression in the Tumor Microenvironment. Adv Cancer Res (2015) 128:95–139. doi: 10.1016/bs.acr.2015.04.002 26216631 PMC4662416

[27] Shackleton EG Ali HY Khan M Pockley GA McArdle SE. Novel Combinatorial Approaches to Tackle the Immunosuppressive Microenvironment of Prostate Cancer. (2021) 13:1145. doi: 10.3390/CANCERS13051145 PMC7962457 33800156

[28] Marigo I Bosio E Solito S Mesa C Fernandez A Dolcetti L. Tumor-Induced Tolerance and Immune Suppression Depend on the C/Ebpβ Transcription Factor. Immunity (2010) 32:790–802. doi: 10.1016/j.immuni.2010.05.010 20605485

[29] Kumar V Patel S Tcyganov E Gabrilovich DI. The Nature of Myeloid-Derived Suppressor Cells in the Tumor Microenvironment. Trends Immunol (2016) 37:1–13. doi: 10.1016/j.it.2016.01.004 26858199 PMC4775398

[30] Gabrilovich DI. Myeloid-Derived Suppressor Cells. Cancer Immunol Res (2017) 5:3–8. doi: 10.1158/2326-6066.CIR-16-0297 28052991 PMC5426480

[31] Grzywa TM Sosnowska A Matryba P Rydzynska Z Jasinski M Nowis D. Myeloid Cell-Derived Arginase in Cancer Immune Response. Front Immunol (2020) 11:938. doi: 10.3389/fimmu.2020.00938 32499785 PMC7242730

[32] Gabrilovich DI Ostrand-Rosenberg S Bronte V. Coordinated Regulation of Myeloid Cells by Tumours. Nat Rev Immunol (2012) 12:253–68. doi: 10.1038/nri3175 PMC3587148 22437938

[33] Hossain DMS Pal SK Moreira D Duttagupta P Zhang Q Won H. TLR9-Targeted STAT3 Silencing Abrogates Immunosuppressive Activity of Myeloid-Derived Suppressor Cells From Prostate Cancer Patients. Clin Cancer Res (2015) 21:3771–82. doi: 10.1158/1078-0432.CCR-14-3145 PMC4537814 25967142

[34] Rébé C Végran F Berger H Ghiringhelli F. STAT3 Activation: A Key Factor in Tumor Immunoescape. JAK-STAT (2013) 2:e23010. doi: 10.4161/JKST.23010 24058791 PMC3670267

[35] de Haas N de Koning C Spilgies L de Vries IJM Hato S V. Improving Cancer Immunotherapy by Targeting the STATe of MDSCs. Oncoimmunology (2016) 5. doi: 10.1080/2162402X.2016.1196312 PMC5006927 27622051

[36] Li BH Garstka MA Li ZF. Chemokines and Their Receptors Promoting the Recruitment of Myeloid-Derived Suppressor Cells Into the Tumor. Mol Immunol (2020) 117:201–15. doi: 10.1016/J.MOLIMM.2019.11.014 31835202

[37] Maynard JP Ertunc O Kulac I Valle JAB-D de Marzo AM Sfanos KS. IL8 Expression Is Associated With Prostate Cancer Aggressiveness and Androgen Receptor Loss in Primary and Metastatic Prostate Cancer. Mol Cancer Res (2020) 18:153–65. doi: 10.1158/1541-7786.MCR-19-0595 31604846

[38] Sanaei MJ Taheri F Heshmati M Bashash D Nazmabadi R Mohammad-Alibeigi F. Comparing the Frequency of CD33 + Pstat3 + Myeloid-Derived Suppressor Cells and IL-17 + Lymphocytes in Patients With Prostate Cancer and Benign Prostatic Hyperplasia. Cell Biol Int (2021) 45:2086–95. doi: 10.1002/CBIN.11651 34184811

[39] Garcia AJ Ruscetti M Arenzana TL Tran LM Bianci-Frias D Sybert E. Pten Null Prostate Epithelium Promotes Localized Myeloid-Derived Suppressor Cell Expansion and Immune Suppression During Tumor Initiation and Progression. Mol Cell Biol (2014) 34:2017. doi: 10.1128/MCB.00090-14 24662052 PMC4019050

[40] Johnson R Halder G. The Two Faces of Hippo: Targeting the Hippo Pathway for Regenerative Medicine and Cancer Treatment. Nat Rev Drug Discovery (2014) 13:63. doi: 10.1038/NRD4161 24336504 PMC4167640

[41] Calcinotto A Spataro C Zagato E di Mitri D Gil V Crespo M. IL-23 Secreted by Myeloid Cells Drives Castration-Resistant Prostate Cancer. (2018) 559:363–9. doi: 10.1038/s41586-018-0266-0 PMC6461206 29950727

[42] Wang G Lu X Dey P Deng P Wu CC Jiang S. Targeting YAP-Dependent MDSCs Infiltration Impairs Tumor Progression. Cancer Discovery (2016) 6:80–95. doi: 10.1158/2159-8290.CD-15-0224 26701088 PMC4707102

[43] Jayaprakash P Ai M Liu A Budhani P Bartkowiak T Sheng J. Targeted Hypoxia Reduction Restores T Cell Infiltration and Sensitizes Prostate Cancer to Immunotherapy. J Clin Invest (2018) 128:5137–49. doi: 10.1172/JCI96268 PMC6205399 30188869

[44] Burkhardt L Fuchs S Krohn A Masser S Mader M Kluth M. CHD1 Is a 5q21 Tumor Suppressor Required for ERG Rearrangement in Prostate Cancer. Cancer Res (2013) 73:2795–805. doi: 10.1158/0008-5472.CAN-12-1342 23492366

[45] Diossy M Tisza V Li H Zhou J Sztupinszki Z Young D. Increased Frequency of CHD1 Deletions in Prostate Cancers of African American Men is Associated With Distinct Homologous Recombination Deficiency Associated DNA Aberration Profiles. medRxiv (2021) 21251199. doi: 10.1101/2021.02.08.21251199

[46] Zhao D Cai L Lu X Liang X Li J Chen P. Chromatin Regulator CHD1 Remodels the Immunosuppressive Tumor Microenvironment in PTEN-Deficient Prostate Cancer. Cancer Discov (2020) 10:1374–87. doi: 10.1158/2159-8290.CD-19-1352 PMC7483306 32385075

[47] Ren W Zhang X Li W Feng Q Feng H Tong Y. Exosomal miRNA-107 Induces Myeloid-Derived Suppressor Cell Expansion in Gastric Cancer. Cancer Manage Res (2019) 11:4023. doi: 10.2147/CMAR.S198886 PMC6511657 31190980

[48] Huber V Vallacchi V Fleming V Hu X Cova A Dugo M. Tumor-Derived microRNAs Induce Myeloid Suppressor Cells and Predict Immunotherapy Resistance in Melanoma. J Clin Invest (2018) 128:5517–30. doi: 10.1172/JCI98060 PMC6264733 30260323

[49] Daveri E Vergani E Shahaj E Bergamaschi L la Magra S Dosi M. microRNAs Shape Myeloid Cell-Mediated Resistance to Cancer Immunotherapy. Front Immunol (2020) 0:1214. doi: 10.3389/FIMMU.2020.01214 PMC7387687 32793185

[50] Cochetti G Poli G Guelfi G Boni A Egidi MG Mearini E. Different Levels of Serum microRNAs in Prostate Cancer and Benign Prostatic Hyperplasia: Evaluation of Potential Diagnostic and Prognostic Role. OncoTargets Ther (2016) 9:7545–53. doi: 10.2147/OTT.S119027 PMC5167485 28008272

[51] Ai L Mu S Wang Y Wang H Cai L Li W. Prognostic Role of Myeloid-Derived Suppressor Cells in Cancers: A Systematic Review and Meta-Analysis. BMC Cancer (2018) 18:1–9. doi: 10.1186/s12885-018-5086-y 30518340 PMC6280417

[52] Zhang X Fu X Li T Yan H. The Prognostic Value of Myeloid Derived Suppressor Cell Level in Hepatocellular Carcinoma: A Systematic Review and Meta-Analysis. PLos One (2019) 14:12:e0225327. doi: 10.1371/journal.pone.0225327 PMC6886785 31790437

[53] Chang G Kang I Shahabi A Nadadur M Athreya K Suer D. MDSCs Clinical Assay for Disease Surveillance in Prostate Cancer. J Clin Oncol (2015) 33:e16091–1. doi: 10.1200/jco.2015.33.15_suppl.e16091

[54] Vuk-Pavlović S Bulur PA Lin Y Qin R Szumlanski CL Zhao X. Immunosuppressive CD14+HLA-DRlow/– Monocytes in Prostate Cancer. Prostate (2010) 70:443–55. doi: 10.1002/PROS.21078 PMC2935631 19902470

[55] Santegoets SJ Stam AG Lougheed SM Gall H Jooss K Sacks N. Myeloid Derived Suppressor and Dendritic Cell Subsets are Related to Clinical Outcome in Prostate Cancer Patients Treated With Prostate GVAX and Ipilimumab. J ImmunoTher Cancer (2014) 2:31. doi: 10.1186/S40425-014-0031-3 26196012 PMC4507359

[56] Takahashi R Amano H Ito Y Eshima K Satoh T Iwamura M. Microsomal Prostaglandin E Synthase-1 Promotes Lung Metastasis *via* SDF-1/CXCR4-Mediated Recruitment of CD11b+Gr1+MDSCs From Bone Marrow. Biomed Pharmacother (2020) 121:109581. doi: 10.1016/J.BIOPHA.2019.109581 31715374

[57] Koga N Moriya F Waki K Yamada A Itoh K Noguchi M. Immunological Efficacy of Herbal Medicines in Prostate Cancer Patients Treated by Personalized Peptide Vaccine. Cancer Sci (2017) 108:2326. doi: 10.1111/CAS.13397 28898532 PMC5715291

[58] Idorn M Køllgaard T Kongsted P Sengeløv L thor Straten P. Correlation Between Frequencies of Blood Monocytic Myeloid-Derived Suppressor Cells, Regulatory T Cells and Negative Prognostic Markers in Patients With Castration-Resistant Metastatic Prostate Cancer. Cancer Immunol Immunother (2014) 63:1177–87. doi: 10.1007/S00262-014-1591-2 PMC11028426 25085000

[59] Lee C-R Kwak Y Yang T Han JH Park S-H Ye MB. Myeloid-Derived Suppressor Cells Are Controlled by Regulatory T Cells *via* TGF-β During Murine Colitis. Cell Rep (2016) 17:3219–32. doi: 10.1016/j.celrep.2016.11.062 28009291

[60] Hellsten R Lilljebjörn L Johansson M Leandersson K Bjartell A. The STAT3 Inhibitor Galiellalactone Inhibits the Generation of MDSCs-Like Monocytes by Prostate Cancer Cells and Decreases Immunosuppressive and Tumorigenic Factors. Prostate (2019) 79:1611–21. doi: 10.1002/PROS.23885 PMC6771992 31348843

[61] Fultang L Panetti S Ng M Collins P Graef S Rizkalla N. MDSCs Targeting With Gemtuzumab Ozogamicin Restores T Cell Immunity and Immunotherapy Against Cancers. EBioMedicine (2019) 47:235–46. doi: 10.1016/j.ebiom.2019.08.025 PMC6796554 31462392

[62] Siemińska I Rychlicka-Buniowska E Jaszczyński J Palaczyński M Bukowska-Strakova K Ryś J. The Level of Myeloid Derived-Suppressor Cells in Peripheral Blood of Patients With Prostate Cancerafter Various Types of Therapy. Polish J Pathol (2020) 71:46–54. doi: 10.5114/PJP.2020.95415 32429654

[63] Trellakis S Bruderek K Hü J Elian M Hoffmann TK Lang S. Granulocytic Myeloid-Derived Suppressor Cells are Cryosensitive and Their Frequency Does Not Correlate With Serum Concentrations of Colony-Stimulating Factors in Head and Neck Cancer. Innate Immun (2013) 19:328–36 doi: 10.1177/1753425912463618 23160385

[64] Grützner E Stirner R Arenz L Athanasoulia AP Schrödl K Berking C. Kinetics of Human Myeloid-Derived Suppressor Cells After Blood Draw. J Trans Med (2016) 14. doi: 10.1186/S12967-015-0755-Y PMC4702395 26733325

[65] Wen J Huang G Liu S Wan J Wang X Zhu Y. Polymorphonuclear MDSCs are Enriched in the Stroma and Expanded in Metastases of Prostate Cancer. J Pathol: Clin Res (2020) 6:171. doi: 10.1002/CJP2.160 32149481 PMC7339199

[66] Lopez-Bujanda ZA Haffner MC Chaimowitz MG Chowdhury N Venturini NJ Obradovic A. Castration-Mediated IL-8 Promotes Myeloid Infiltration and Prostate Cancer Progression. bioRxiv (2019) 651083. doi: 10.1101/651083 PMC9169571 35122025

[67] Zhiguang X Lerman I Hammes SR. SAT-138 Neutrophil Elastase Promotes Proliferative Signals in Prostate Cells Through EGFR and DDR1. J Endocr Soc (2020) 4:1. doi: 10.1210/jendso/bvaa046.140

[68] Lerman I de la Luz Garcia-Hernandez M Rangel-Moreno J Chiriboga L Pan C Nastiuk KL. Infiltrating Myeloid Cells Exert Protumorigenic Actions *via* Neutrophil Elastase. Mol Cancer Res (2017) 15:1138–52. doi: 10.1158/1541-7786.MCR-17-0003 PMC5581693 28512253

[69] Bruchard M Mignot G Derangère V Chalmin F Chevriaux A Végran F. Chemotherapy-Triggered Cathepsin B Release in Myeloid-Derived Suppressor Cells Activates the Nlrp3 Inflammasome and Promotes Tumor Growth. Nat Med (2012) 19:57–64. doi: 10.1038/nm.2999 23202296

[70] Kaur P Asea A. Radiation-Induced Effects and the Immune System in Cancer. Front Oncol (2012) 0:191. doi: 10.3389/FONC.2012.00191 PMC3523399 23251903

[71] Lin L Kane N Kobayashi N Kono EA Yamashiro JM Nickols NG. High-Dose Per Fraction Radiotherapy Induces Both Antitumor Immunity and Immunosuppressive Responses in Prostate Tumors. Clin Cancer Res (2021) 27:1505–15. doi: 10.1158/1078-0432.CCR-20-2293 33219015

[72] May KF Jr. Gulley JL Drake CG Dranoff G Kantoff PW. Prostate Cancer Immunotherapy. Clin Cancer Res (2011) 17:5233. doi: 10.1158/1078-0432.CCR-10-3402 21700764 PMC3263933

[73] Chen F Zhao X. Prostate Cancer: Current Treatment and Prevention Strategies. Iranian Red Crescent Med J (2013) 15:279. doi: 10.5812/IRCMJ.6499 PMC3785898 24082997

[74] Poeta VM Massara M Capucetti A Bonecchi R. Chemokines and Chemokine Receptors: New Targets for Cancer Immunotherapy. Front Immunol (2019) 10:379. doi: 10.3389/FIMMU.2019.00379 30894861 PMC6414456

[75] Koivisto CS Parrish M Bonala SB Ngoi S Torres A Gallagher J. Evaluating the Efficacy of Enzalutamide and the Development of Resistance in a Preclinical Mouse Model of Type-I Endometrial Carcinoma. bioRxiv (2019). doi: 10.1101/2019.12.06.86818 PMC7452078 32818842

[76] Kwilas AR Ardiani A Donahue RN Aftab DT Hodge JW. Dual Effects of a Targeted Small-Molecule Inhibitor (Cabozantinib) on Immune-Mediated Killing of Tumor Cells and Immune Tumor Microenvironment Permissiveness When Combined With a Cancer Vaccine. J Transl Med (2014) 12:1–15. doi: 10.1186/s12967-014-0294-y 25388653 PMC4236498

[77] Lesinski GB Reville PK Mace TA Young GS Ahn-Jarvis J Thomas-Ahner J. Consumption of Soy Isoflavone Enriched Bread in Men With Prostate Cancer Is Associated With Reduced Proinflammatory Cytokines and Immunosuppressive Cells. Cancer Prev Res (Phila) (2015)8:1036–44. doi: 10.1158/1940-6207.CAPR-14-0464 PMC4633400 26276751

[78] Moreira D Adamus T Zhao X Su YL Zhang Z White SV. STAT3 Inhibition Combined With CpG Immunostimulation Activates Antitumor Immunity to Eradicate Genetically Distinct Castration-Resistant Prostate Cancers. Clin Cancer Res (2018) 24:5948–62. doi: 10.1158/1078-0432.CCR-18-1277 PMC6279477 30337279

[79] Kumar V Cheng P Condamine T Mony S Languino LR McCaffrey JC. CD45 Phosphatase Inhibits STAT3 Transcription Factor Activity in Myeloid Cells and Promotes Tumor-Associated Macrophage Differentiation. Immunity (2016) 44:303. doi: 10.1016/J.IMMUNI.2016.01.014 26885857 PMC4759655

[80] Zhou J Shen Q Lin H Hu L Li G Zhang X. Decitabine Shows Potent Anti-Myeloma Activity by Depleting Monocytic Myeloid-Derived Suppressor Cells in the Myeloma Microenvironment. J Cancer Res Clin Oncol (2019) 145:329–36. doi: 10.1007/s00432-018-2790-6 PMC11810327 30426212

[81] Tu SP Jin H Shi JD Zhu LM Suo Y Lu G. Curcumin Induces the Differentiation of Myeloid-Derived Suppressor Cells and Inhibits Their Interaction With Cancer Cells and Related Tumor Growth. Cancer Prev Res (2012) 5:205–15. doi: 10.1158/1940-6207.CAPR-11-0247 PMC3273601 22030090

[82] Murphy EJ Rezoagli E Major I Rowan NJ Laffey JG. β-Glucan Metabolic and Immunomodulatory Properties and Potential for Clinical Application. J Fungi (2020) 10:356. doi: 10.3390/jof6040356 PMC7770584 33322069

[83] Albeituni SH Ding C Liu M Hu X Luo F Kloecker G. Yeast-Derived Particulate β-Glucan Treatment Subverts the Suppression of Myeloid-Derived Suppressor Cells (MDSCs) by Inducing Polymorphonuclear MDSCs Apoptosis and Monocytic MDSCs Differentiation to APC in Cancer. J Immunol (2016) 196:2167–80. doi: 10.4049/jimmunol.1501853 PMC4761495 26810222

[84] Twardowski P Kanaya N Frankel P Synold T Ruel C Pal SK. A Phase I Trial of Mushroom Powder in Patients With Biochemically Recurrent Prostate Cancer: Roles of Cytokines and Myeloid-Derived Suppressor Cells for Agaricus Bisporus-Induced Prostate-Specific Antigen Responses. Cancer (2015) 121:2942–50. doi: 10.1002/cncr.29421 PMC5685188 25989179

[85] Tran C Ouk S Clegg NJ Chen Y Watson PA Arora V. Development of a Second-Generation Antiandrogen for Treatment of Advanced Prostate Cancer. Science (2009) 324:787–90. doi: 10.1126/SCIENCE.1168175 PMC2981508 19359544

[86] Weber R Fleming V Hu X Nagibin V Groth C Altevogt P. Myeloid-Derived Suppressor Cells Hinder the Anti-Cancer Activity of Immune Checkpoint Inhibitors. (2018) 9:1. doi: 10.3389/fimmu.2018.01310 PMC6004385 29942309

[87] Le HK Graham L Cha E Morales JK Manjili MH Bear HD. Gemcitabine Directly Inhibits Myeloid Derived Suppressor Cells in BALB/c Mice Bearing 4T1 Mammary Carcinoma and Augments Expansion of T Cells From Tumor-Bearing Mice. Int Immunopharmacol (2009) 9:900–9. doi: 10.1016/J.INTIMP.2009.03.015 19336265

[88] Lu W Yu W He J Liu W Yang J Lin X. Reprogramming Immunosuppressive Myeloid Cells Facilitates Immunotherapy for Colorectal Cancer. EMBO Mol Med (2021) 13:e12798. doi: 10.15252/EMMM.202012798 33283987 PMC7799360

[89] Shen XZ Okwan-Duodu D Blackwell WL Ong FS Janjulia T Bernstein EA. Myeloid Expression of Angiotensin-Converting Enzyme Facilitates Myeloid Maturation and Inhibits the Development of Myeloid-Derived Suppressor Cells. Lab Invest (2014) 94:536–44. doi: 10.1038/LABINVEST.2014.41 PMC4221240 24614194

[90] Domińska K Okła P Kowalska K Habrowska-Górczyńska DE Urbanek KA Ochędalski T. Angiotensin 1–7 Modulates Molecular and Cellular Processes Central to the Pathogenesis of Prostate Cancer. Sci Rep (2018) 8:1–12. doi: 10.1038/s41598-018-34049-8 30361641 PMC6202343

[91] Domińska K Kowalska K Anna Urbanek K Ewa Habrowska-Górczy D Och T Wanda Piastowska Ciesielska A. The Impact of Ang-(1-9) and Ang-(3-7) on the Biological Properties of Prostate Cancer Cells by Modulation of Inflammatory and Steroidogenesis Pathway Genes. Int J Mol Sci Article (2020) 21:6227. doi: 10.3390/ijms21176227 PMC7504072 32872192

[92] Ockrim J Lalani EN Abel P. Therapy Insight: Parenteral Estrogen Treatment for Prostate Cancer - A New Dawn for an Old Therapy. Nat Clin Pract Oncol (2006) 3:552–63. doi: 10.1038/NCPONC060 17019433

[93] Huang S Zhou N Zhao L Gimple RC Ahn YH Zhang P. Pharmacological Activation of Estrogen Receptor Beta Overcomes Tumor Resistance to Immune Checkpoint Blockade Therapy. iScience (2020) 23:101458. doi: 10.1016/J.ISCI.2020.101458 32861994 PMC7476860

[94] di Zazzo E Galasso G Giovannelli P di Donato M Castoria G. Estrogens and Their Receptors in Prostate Cancer: Therapeutic Implications. Front Oncol (2018) 8:2. doi: 10.3389/FONC.2018.00002 29404276 PMC5778111

[95] Gehrig J Kaulfuß S Jarry H Bremmer F Stettner M Burfeind P. Prospects of Estrogen Receptor β Activation in the Treatment of Castration-Resistant Prostate Cancer. Oncotarget (2017) 8:34971–9. doi: 10.18632/ONCOTARGET.16496 PMC5471027 28380417

[96] Sanaei MJ Salimzadeh L Bagheri N. Crosstalk Between Myeloid-Derived Suppressor Cells and the Immune System in Prostate Cancer: MDSCs and Immune System in Prostate Cancer. J Leukoc Biol (2020) 107:43–56. doi: 10.1002/JLB.4RU0819-150RR 31721301

[97] Ollivier L Labbé M Fradin D Potiron V Supiot S. Interaction Between Modern Radiotherapy and Immunotherapy for Metastatic Prostate Cancer. Front Oncol (2021) 11:744679/BIBTEX. doi: 10.3389/FONC.2021.744679/BIBTEX 34595122 PMC8477651

